# Genetically modified plants are an alternative to oily fish for providing *n*‐3 polyunsaturated fatty acids in the human diet: A summary of the findings of a Biotechnology and Biological Sciences Research Council funded project

**DOI:** 10.1111/nbu.12478

**Published:** 2020-12-23

**Authors:** A. L. West, E. A. Miles, K. A. Lillycrop, J. A. Napier, P. C. Calder, G. C. Burdge

**Affiliations:** ^1^ Faculty of Medicine School of Human Development and Health University of Southampton Southampton UK; ^2^ Faculty of Natural and Environmental Sciences Centre for Biological Sciences University of Southampton Southampton UK; ^3^ Department of Plant Sciences Rothamsted Research Harpenden UK; ^4^ NIHR Southampton Biomedical Research Centre University Hospital Southampton NHS Foundation Trust University of Southampton Southampton UK

**Keywords:** human diet, nutrition, omega‐3 fatty acids, polyunsaturates, transgenic plant

## Abstract

The *n*‐3 polyunsaturated fatty acids (PUFA) present primarily in oily fish, namely eicosapentaenoic acid (EPA) and docosahexaenoic acid (DHA), are important components of cell membranes and that are needed for normal development and cell function. Humans have very limited capacity for EPA and DHA synthesis from α‐linolenic acid and so they must be obtained pre‐formed from the diet. However, perceived unpalatability of oily fish and fish oil concerns about contamination with environmental pollutants, dietary choices that exclude fish and animal products, and price limit the effectiveness of recommendations for EPA and DHA intakes. Moreover, marine sources of EPA and DHA are diminishing in the face of increasing demands. Therefore, an alternative source of EPA and DHA is needed that is broadly acceptable, can be upscaled and is sustainable. This review discusses these challenges and, using findings from recent nutritional trials, explains how they may be overcome by seed oils from transgenic plants engineered to produce EPA and DHA. Trials in healthy men and women assessed the acute uptake and appearance in blood over 8 hours of EPA and DHA from transgenic *Camelina sativa* compared to fish oil, and the incorporation of these PUFA into blood lipids after dietary supplementation. The findings showed that postprandial EPA and DHA incorporation into blood lipids and accumulation in plasma lipids after dietary supplementation was as good as that achieved with fish oil. The oil derived from this transgenic plant was well tolerated. This review also discusses the implications for human nutrition, marine ecology and agriculture.

## *n*‐3 polyunsaturated fatty acids in tissues and in the diet

*n*‐3 (also known as omega‐3) polyunsaturated fatty acids (PUFA) are a group of fatty acids in which the first double bond is three carbons from the methyl end of the molecule. In humans, *n*‐3 PUFA in the diet and in cell membranes typically vary in the length of the hydrocarbon chain between 18 and 22 carbons, and in the degree of unsaturation between three and six double bonds, all in the *cis* configuration (Burdge & Calder 2015). The four principle *n*‐3 PUFA are unevenly distributed between tissues (Arterburn *et al*. 2006), which suggests that their incorporation into cell membranes is related to specific cell functions. α‐Linolenic acid (18:3*n*‐3, ALA) and eicosapentaenoic acid (20:5*n*‐3, EPA) each account for less than 1% of total membrane fatty acids in human tissues (Arterburn *et al*. 2006). In contrast, docosahexaenoic acid (22:6*n*‐3, DHA) accounts for over 20% of fatty acids in the retina and more than 15% of total sperm and cerebral cortex fatty acids (Arterburn *et al*. 2006). Longer chain, more unsaturated *n*‐3 PUFA also exist as metabolic intermediates that do not accumulate in cells, for example, 24:6*n*‐3 in the Sprecher pathway (Leonard *et al*. 2004), and in specialised membrane structures, such as lipid rafts, which contain *n*‐3 PUFA with chain length ≥24 carbons (Vasireddy *et al*. 2007).

ALA is the only omega‐3 PUFA that is considered to be essential in the human diet (Baker *et al*. 2016), although DHA may be conditionally essential during pregnancy (Larque *et al*. 2002). ALA is found in a number of seeds and seed oils, such as walnut oil, soybean oil and flaxseed oil, in which ALA contributes approximately 50% of the seed oil fatty acids. Typical intakes of ALA in European countries, North America and Australia range from 0.5 to 2.5 g/day (Ollis *et al*. 1999; Kris‐Etherton *et al*. 2000; Burdge & Calder 2005a). EPA, docosapentaenoic acid *n*‐3 (22:5*n*‐3, DPA*n*‐3) and DHA are absent from plants; the main dietary sources of these fatty acids are oily fish, such as salmon, sardines and mackerel, and their food products (Monroig *et al*. 2018) and, albeit in markedly lower amounts, meat and dairy products (Dugan *et al*. 2018).

## Omega‐3 polyunsaturated fatty acids and human health

There is a robust literature that demonstrates that adequate consumption of *n*‐3 PUFA derived from marine fish, specifically EPA and DHA, are important for maintaining health across the life course. For example, EPA + DHA status (Albert *et al*. 2002) and dietary intake (He *et al*. 2004) have been shown to improve cardiovascular health and pharmaceutical preparations of these fatty acids have been demonstrated in meta‐analyses to be as effective as statins in preventing myocardial infarction (Studer *et al*. 2005), with inconsistencies in outcomes between studies likely to be attributable to limitations in the study designs (Calder & Yaqoob 2012). Increased EPA plus DHA intakes can also improve chronic inflammatory and allergic diseases such as rheumatoid arthritis (Miles & Calder 2012), asthma and inflammatory bowel disease (Calder 2015). Such beneficial effects on health are mediated via changes in the structure and function of cell membranes (both lipid and protein components) and by altering the profile of secreted eicosanoids and specialised pro‐resolving lipid mediators (Thies *et al*. 2003; Calder 2012; Calder 2015). Furthermore, adequate accumulation of DHA into neural membranes is important for the normal development of the retina and central nervous system (Carlson & Neuringer 1999; Lauritzen *et al*. 2001).

In recognition of these positive impacts on health, several regulatory/advisory bodies have made recommendations for intakes of EPA plus DHA ranging from 250 to 1000 mg/day in order to promote optimal health. In the UK, the Scientific Advisory Committee on Nutrition (SACN) recommended that all adults should consume at least 450 mg/day of EPA plus DHA (SACN 2004). However, the effectiveness of such recommendations in promoting health is limited by dietary choices and the sustainability of the supply of marine fish as a primary source of these fatty acids.

## Challenges to optimal EPA and DHA intakes in the UK population

Despite the strength of the evidence in support of the health benefits derived from regular consumption of EPA and DHA, habitual intakes of these fatty acids across the UK population are disappointingly low. Total fish consumption in the UK has decreased by approximately 25% since 1950, and only 27% of adult fish consumers regularly eat oily fish (Givens & Gibbs 2006). Consequently, most UK adults consume less than 50% of the amount of EPA plus DHA that is recommended to maintain health. EPA plus DHA intake in children is approximately only 10% of that in adults (Givens & Gibbs 2008). Although cost of oily fish may be a concern, the perceived unpalatability of oily fish also appears to be a major barrier to its consumption (Givens & Gibbs 2008). Approximately 11% of the UK population regularly consume EPA plus DHA as a fish oil dietary supplement, which is most common in individuals aged over 55 years (Food Standards Agency 2008). The use of fish oil supplements is also limited by perceived unpalatability, cost and concerns about contamination with pollutants. Furthermore, inconsistencies have been identified in the quality of fish oil supplements (Albert *et al*. 2015) although others have not found this to be the case (Nichols *et al*. 2016; Bannenberg *et al*. 2017). Dietary choices that exclude meat, dairy products and/or fish induce lower EPA and DHA status (Sanders 2009; Burdge *et al*. 2017), which is not offset by metabolic adaptations during pregnancy (Postle *et al*. 1995), such that breastmilk and infant blood EPA and DHA have been reported to be approximately 50% lower in vegetarians and vegans, compared to omnivores (Sanders & Reddy 1992). However, despite the importance of DHA as a component of neural membranes, neither vegetarian dietary choice (Crozier *et al*. 2019) nor maternal DHA status (Crozier *et al*. 2018) in pregnancy appears to affect cognitive function in children.

## α‐Linolenic acid is not an effective alternative to pre‐formed EPA and DHA in the diet

EPA and DHA can be synthesised by some mammals, in particular rodents, from ALA (Voss *et al*. 1991). This finding has underpinned a number of studies that have sought to test whether ALA is, through metabolic interconversion, an effective alternative source of EPA and DHA to consuming these PUFA pre‐formed. If that were the case, this would address at least some of the major barriers to achieving recommended EPA and DHA intakes, in particular cost, palatability and exclusion of animal‐derived foods from the diet. However, the results of dietary intervention trials that increased ALA intake (Burdge & Calder 2006; Baker *et al*. 2016) and of stable isotope tracer studies involving labelled ALA (Burdge 2004) have shown that the activity of this pathway is low in humans such that capacity for EPA synthesis is limited in men and women (Burdge & Wootton 2002; Burdge *et al*. 2002) and there is no evidence of DHA synthesis in men (Burdge *et al*. 2002). However, women are able to synthesise DHA (Burdge & Wootton 2002) and maintain higher DHA status than men (Lohner *et al*. 2013) irrespective of their background diet (Welch *et al*. 2010). This sex difference in capacity for DHA synthesis and DHA status appears to be lost in older individuals (Pittaway *et al*. 2015) who, therefore, are likely to have a greater reliance on consuming pre‐formed EPA + DHA than younger people. Thus, increased intake of ALA does not provide an alternative dietary source of EPA and DHA to consuming these PUFA pre‐formed (Burdge & Calder 2006). Moreover, dietary supplementation with stearidonic acid (18:4*n*‐3, SDA), the product of the initial rate‐limiting reaction in hepatic conversion of ALA to EPA and DHA, has been shown to increase EPA and DPA*n*‐3 status, but not that of DHA (James *et al*. 2003). Increased consumption of SDA, which is only present in significant amounts in members of the genus Echium (Guil‐Guerrero *et al*. 2001), is also an unsuitable dietary strategy for increasing EPA + DHA levels. Overall, these findings show that plant‐derived *n*‐3 fatty acids, specifically ALA and SDA, are not effective replacements for pre‐formed EPA and DHA in the human diet (Plourde & Cunnane 2007).

## Meeting global demands for EPA and DHA is a major burden on marine ecology

There is an emerging consensus that a daily intake of approximately 500 mg EPA + DHA per individual would confer health benefits, which Salem and Eggersdorfer (2015) estimate would require 1.3 million metric tonnes annually of EPA plus DHA for the seven billion humans that inhabit the Earth. Currently, marine sources can supply 0.2 million metric tonnes of EPA plus DHA; about 16% of the required global provision of EPA plus DHA (Salem & Eggersdorfer 2015). Wild fish capture has remained static since the 1990s, although the production of farmed fish has increased exponentially during this period. However, aquaculture also represents a major demand on world fish oil supply as this is required for rearing of many farmed species (Tocher 2015). There is also increasing demand for highly purified EPA and DHA for preparation of ethyl ester‐based pharmaceutical products which incurs high production losses (90–95%; Kitessa *et al*. 2014).

Thus, there is a critical need for novel, sustainable and scalable sources of EPA and DHA in order to ensure sufficient supply for the global population.

### Crustacea and algal sources of pre‐formed EPA and DHA

Krill oil has been proposed as an alternative to oily fish as a source of EPA plus DHA. However, the estimated yield from the total krill catch in 2013 was 625 metric tonnes, about 0.3% of the global EPA + DHA requirement (Kwantas & Grundmann 2015). Although this could be increased to 9% with considerable investment, this would have implications for the conservation and stability of marine food webs in the South Atlantic (Kwantas & Grundmann 2015). Furthermore, claims for greater bioavailability of *n*‐3 PUFA from krill oil compared to fish oil have been shown to be unfounded (Salem & Kuratko 2014). Algal oils that contain DHA or EPA plus DHA have also been proposed as an alternative source of these fatty acids (Kuratko *et al*. 2013) and these currently account for 2% of human EPA + DHA consumption (Salem & Eggersdorfer 2015). However, the current costs of fermentation and refining processes are substantially greater than for fish oil production and hence algal oils are not likely to be economically competitive in the medium term as a replacement for oily fish in the human diet (Salem & Eggersdorfer 2015).

## Oils from genetically modified plants as a source of EPA and DHA

Vegetable oils derived from genetically modified plants represent a potentially attractive alternative source of EPA and DHA for human consumption since such oils may overcome the concerns about palatability, sustainability and contamination that are associated with fish oil, the limited conversion of ALA in humans, and the cost and conservation issues associated with algal oils and krill oil, respectively. Importantly, the health benefits derived from fish oil are due to both EPA and DHA although their functions may differ (Innes & Calder 2018), and thus, oils from transgenic plants that only contain one of these *n*‐3 PUFA may have limited effectiveness in supporting optimal health compared to consuming both pre‐formed EPA and DHA.

**Figure 1:**
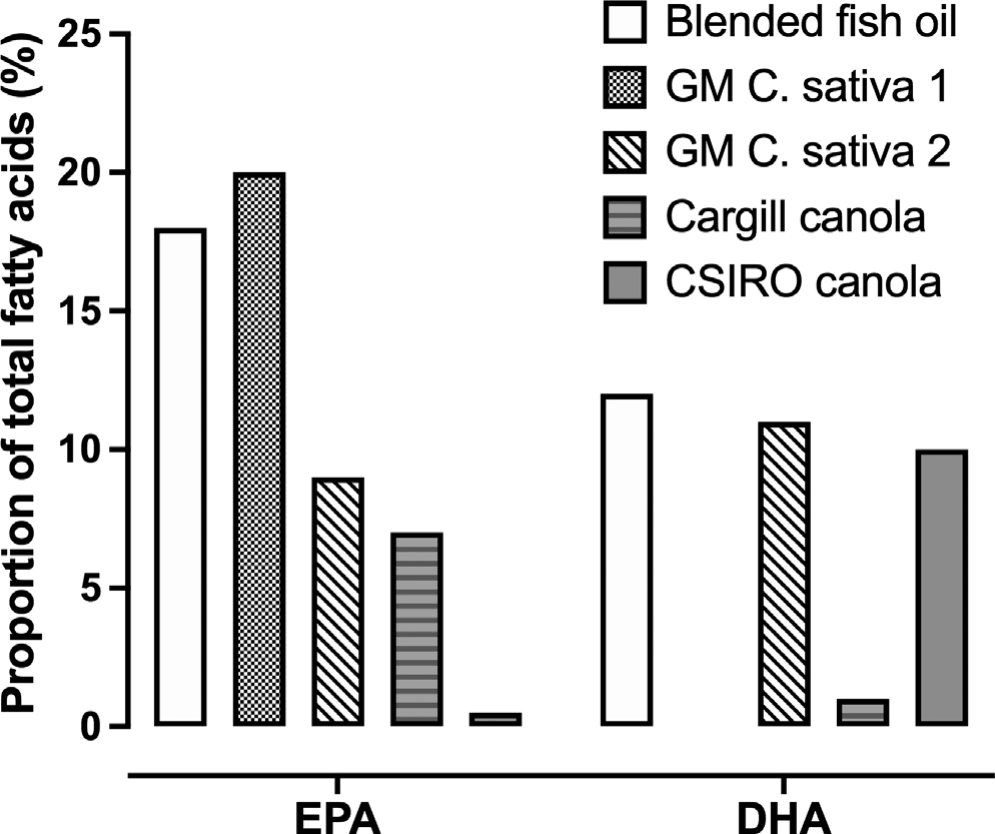
Proportions of eicosapentaenoic acid (EPA) and docosahexaenoic acid (DHA) in fish oil and in oils from transgenic (GM) plants that were engineered to synthesise EPA and DHA. Values from Tocher *et al*. (2019) and West *et al*. (2020a).

There are no native land plant species which can synthesise EPA and DHA. However, genetic insertion of desaturase and elongase enzymes from yeast and algae into plants that naturally produce ALA has facilitated the development of strains of canola (oilseed rape) and *Camelina sativa* (*C. sativa*) which produce oils that contain either EPA or DHA, or EPA plus DHA in proportions that approximate, to varying extents the EPA and DHA contents of at least some fish oils (Fig. 1; Napier *et al*. 2019; Han *et al*. 2020). A transgenic strain of canola that produces an oil containing 10% DHA, but less than 1% EPA has been developed by the Commonwealth Scientific and Industrial Research Organisation (Petrie *et al*. 2020) and commercialised by Nuseed (Tocher *et al*. 2019). Similarly, a transgenic strain of canola that produces a seed oil which contains 0.2% DHA and 8.1% EPA has been commercialised by Cargill for aquaculture feed (Napier *et al*. 2019). However, as far as we are aware, there is no information in the public domain that reports testing of the oils produced by transgenic strains of canola for their effectiveness in replacing fish oil as a source of EPA and DHA in the human diet.

## Effectiveness of transgenic *Camelina sativa* oil compared to fish oil on EPA and DHA uptake and incorporation into blood lipids

**Figure 2:**
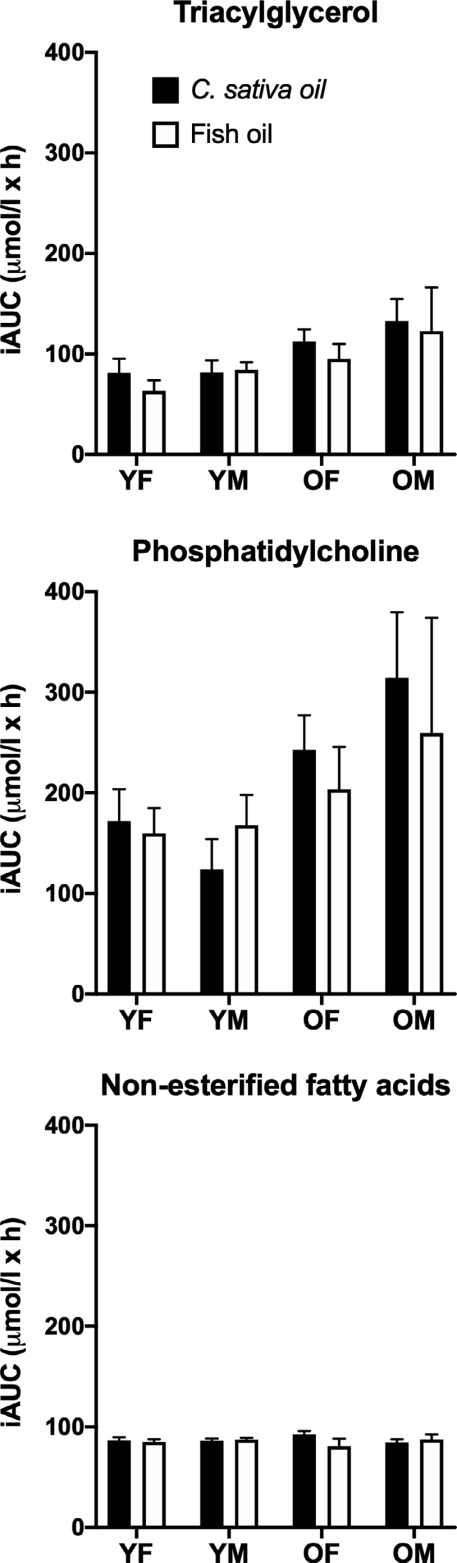
Postprandial incorporation of eicosapentaenoic acid (EPA) and docosahexaenoic acid (DHA) into plasma lipids when consumed as blended fish oil or transgenic *C. sativa* oil. Values are mean ± SEM incremental area under the curve (iAUC) concentrations of EPA plus DHA over 8 hours in plasma. YF, young females; YM, young males; OF, older females; OM, older males. *n* = 10 participants per group. Data are from West *et al*. (2019).

A recent study funded by the Biotechnology and Biological Sciences Research Council UK has conducted the first evaluation in humans of the effectiveness as a source of EPA and DHA of an oil from a transgenic plant that produces EPA and DHA compared to fish oil (West *et al*. 2019, 2020a,b). The study tested the seed oil from transgenic *C. sativa* that contained 11% EPA and 9% DHA (Ruiz‐Lopez *et al*. 2014), which had been shown to be effective in increasing EPA and DHA concentrations in farmed salmon and sea bream (Betancor *et al*. 2017, 2016). The triacylglycerol (TAG) molecular species composition of the transgenic oil differed substantially from the commercially prepared blended fish oil which was used as a comparator (West *et al*. 2020b). In order to test whether such differences in the composition of the oils affected absorption and appearance in blood, the EPA and DHA incorporation into plasma lipids was assessed over an 8‐hour postprandial period. Participants were healthy adults with mean (± standard error of the mean) ages 25 ± 1 years (both younger men and women), 57 ± 1 years (older women) and 60 ± 2 years (older men). The study had a double‐blinded, cross‐over design in which participants consumed a standardised breakfast that included 450 mg EPA plus DHA, which reflects the intake of EPA plus DHA recommended by the UK government (SACN 2004), provided by either blended fish oil or transgenic *C. sativa* oil, and incorporation into blood lipids was then followed over 8 hours (West *et al*. 2019). There were no significant differences in the incremental area under the time versus concentration curve between the test oils in the incorporation of EPA and DHA into plasma TAG, phosphatidylcholine (PC) or non‐esterified fatty acids (NEFA) during the postprandial period (Fig. 2). There was no statistically significant effect of sex on the incorporation of EPA and DHA into these lipid pools. Incorporation of EPA and DHA into plasma TAG, PC and NEFA was approximately 40–60% greater in older subjects compared to the younger participants, although there were no statistically significant interaction effects of age, sex and test oil.

The postprandial period is associated with increased concentrations in blood of specific pro‐inflammatory cytokines (Burdge & Calder 2005b). The findings of West *et al*. (2019) showed no significant difference between test oils in the postprandial changes in the concentrations of TNFα, interleukin (IL)‐6 or IL‐10 and the soluble intercellular adhesion molecule‐1 (West *et al*. 2019). Since the anti‐inflammatory effect of EPA and DHA is well established (Miles & Calder 2012), one possible implication of this finding is that the transgenic *C. sativa* oil may suppress inflammation in a similar extent and manner to fish oil. Fish oil has been suggested to potentially have utility in the management of patients with severe, acute inflammatory diseases such as sepsis (Hall *et al*. 2016) and COVID‐19 (Bistrian 2020; Torrinhas *et al*. 2020). Hence, it is possible that transgenic *C. sativa* oil may also have therapeutic value in such patients.

**Figure 3:**
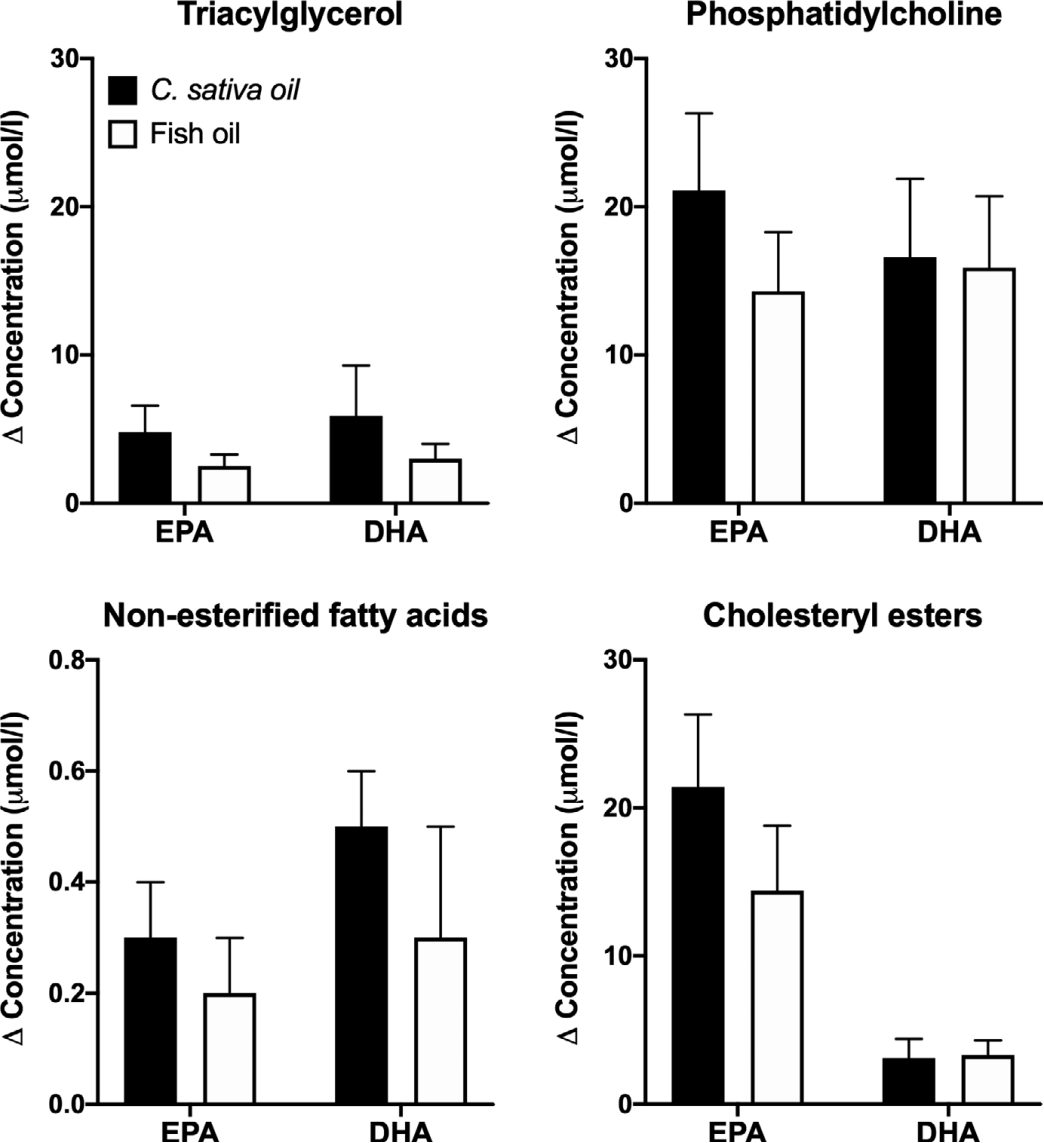
Change from baseline of eicosapentaenoic acid (EPA) and docosahexaenoic acid (DHA) concentrations in plasma lipids when consumed as blended fish oil or transgenic *C. sativa* oil. Values are mean ± SEM increments in EPA and DHA concentrations after dietary supplementation with these test oils for 8 weeks. *n* = 31 participants. Data are from West *et al.* (2020a).

In order to test whether this transgenic *C. sativa* oil was as effective as fish oil in increasing plasma lipid EPA and DHA concentrations over the longer term, the same group carried out an 8‐week, single‐blinded, cross‐over, dietary supplementation trial in a combined group of healthy men and women aged between 20 and 74 years (median 53 years; West *et al*. 2020a). Participants consumed 450 mg/day EPA plus DHA provided as either transgenic *C. sativa* oil or blended fish oil using oral dosing syringes for 8 weeks, followed by 6 weeks washout before consuming the other oil for 8 weeks. Median compliance was greater than 95% for both test oils. Consuming either test oil increased EPA and DHA concentrations in plasma TAG, PC and NEFA, and there were no significant differences between test oils in the magnitude of the increment in plasma EPA and DHA concentrations (Fig. 3). The concentration of TAG in a combined VLDL + chylomicron fraction was reduced significantly and to a similar extent after consuming fish oil (−15%) or transgenic *C. sativa* oil (−11.7%), although there was no significant effect of either oil on total plasma TAG concentration. Moreover, consuming either oil increased the omega‐3 index (Harris & Von Schacky 2004) by approximately 0.6 % points (West *et al*. 2020a). However, the study was not powered to detect changes in these secondary outcomes and so the findings should be regarded as indicative of the potential for health benefits from consuming the transgenic *C. sativa* oil rather than as evidence of efficacy.

The blended fish oil and the transgenic *C. sativa* oil were well tolerated in both trials with no adverse events that could be attributed to consuming either test oil. However, one participant withdrew from the dietary supplementation trial because they found the fish oil supplement unpalatable.

## Conclusions and future perspectives

The recent studies that tested the efficacy of transgenic *C. sativa* oil as a source of EPA and DHA for human consumption indicate that this oil is an effective alternative to fish oil. One important implication of these findings is that this oil could potentially overcome the present challenges to achieving adequate intakes of EPA and DHA in the general population; namely perceived unpalatability and incompatibility with dietary choices that exclude food groups that contain EPA and DHA including meat, oily fish and dairy products. Moreover, the production of transgenic *C. sativa* can potentially be scaled to meet demands (Han *et al*. 2020) in a manner that could not be readily achieved by costly fermentation of algae or wild capture of oily fish, hence reducing the negative impact on the marine environment of producing EPA and DHA for direct human consumption and for aquaculture.

Field trials have demonstrated the stability and robustness of the transgenic trait of *C. sativa* that produces EPA and DHA (Han *et al*. 2020), thus further supporting the sustainability and scalability of this crop which, therefore, represents a novel opportunity for agricultural innovation and commercialisation. However, in some countries, most notably the EU, legislation impeding the commercial cultivation of transgenic plants for human consumption remains a barrier to realisation of such benefits. With the UK’s departure from the EU, it will be interesting to see whether new opportunities for the production of such transgenic crops emerge, although cultivation in parts of the world with fewer legislative restraints (such as North and South America) is a more immediately tangible opportunity. One key development objective is to formulate the means of incorporating the seed oil from transgenic *C. sativa* that produces EPA and DHA into the human food chain in a manner which is cost‐effective, preserves the biologically important PUFA, allows wide consumption and is compatible with most dietary choices.

Overall, transgenic *C. sativa* that produces EPA and DHA could potentially make a significant contribution to facilitating nutrient security with respect to *n*‐3 PUFA while reducing the burden on the marine environment of sourcing these fatty acids for human consumption.

## Conflict of interest

The authors declare no conflicts of interest with this review.

## Author contributions

GCB wrote the first draft of the manuscript with contributions from all authors. All authors approved the final version.

## Declarations

GCB has served as member of the Scientific Advisory Board of BASF AS and as a member of the BASF Newtrition^®^ Asia Research Grant Panel. GCB and KAL have received research funding from Nestlé, Abbott Nutrition and Danone. PCC acts as a consultant to BASF AS, Smartfish, DSM, Cargill and Fresenius‐Kabi. JAN has provided *ad hoc* consultancy services to BASF. The other authors have nothing to declare.
